# Genetic diversity in centipedegrass [*Eremochloa ophiuroides* (Munro) Hack.]

**DOI:** 10.1038/s41438-019-0228-1

**Published:** 2020-01-01

**Authors:** Jianjian Li, Hailin Guo, Junqin Zong, Jingbo Chen, Dandan Li, Jianxiu Liu

**Affiliations:** 1 The National Forestry and Grassland Administration Engineering Research Center for Germplasm Innovation and Utilization of Warm-season Turfgrasses, Institute of Botany, Jiangsu Province and Chinese Academy of Sciences, Nanjing Botanical Garden, Mem. Sun Yat-Sen, 210014 Nanjing, Jiangsu China; 2 The Jiangsu Provincial Engineering and Technology Research Center for Turf Germplasm Improvement and Breeding, Institute of Botany, Jiangsu Province and Chinese Academy of Sciences, Nanjing Botanical Garden, Mem. Sun Yat-Sen, 210014 Nanjing, Jiangsu China

**Keywords:** Plant genetics, Plant genetics

## Abstract

Genetic diversity is the heritable variation within and among populations, and in the context of this paper describes the heritable variation among the germplasm resources of centipedegrass. Centipedegrass is an important warm-season perennial C_4_ grass belonging to the Poaceae family in the subfamily Panicoideae and genus *Eremochloa*. It is the only species cultivated for turf among the eight species in *Eremochloa*. The center of origin for this species is southern to central China. Although centipedegrass is an excellent lawn grass and is most widely used in the southeastern United States, China has the largest reserve of centipedegrass germplasm in the world. Presently, the gene bank in China holds ~200 centipedegrass accessions collected from geographical regions that are diverse in terms of climate and elevation. This collection appears to have broad variability with regard to morphological and physiological characteristics. To efficiently develop new centipedegrass varieties and improve cultivated species by fully utilizing this variability, multiple approaches have been implemented in recent years to detect the extent of variation and to unravel the patterns of genetic diversity among centipedegrass collections. In this review, we briefly summarize research progress in investigating the diversity of centipedegrass using morphological, physiological, cytological, and molecular biological approaches, and present the current status of genomic studies in centipedegrass. Perspectives on future research on genetics and genomics and modern breeding of centipedegrass are also discussed.

## Introduction

**Fig. 1:**
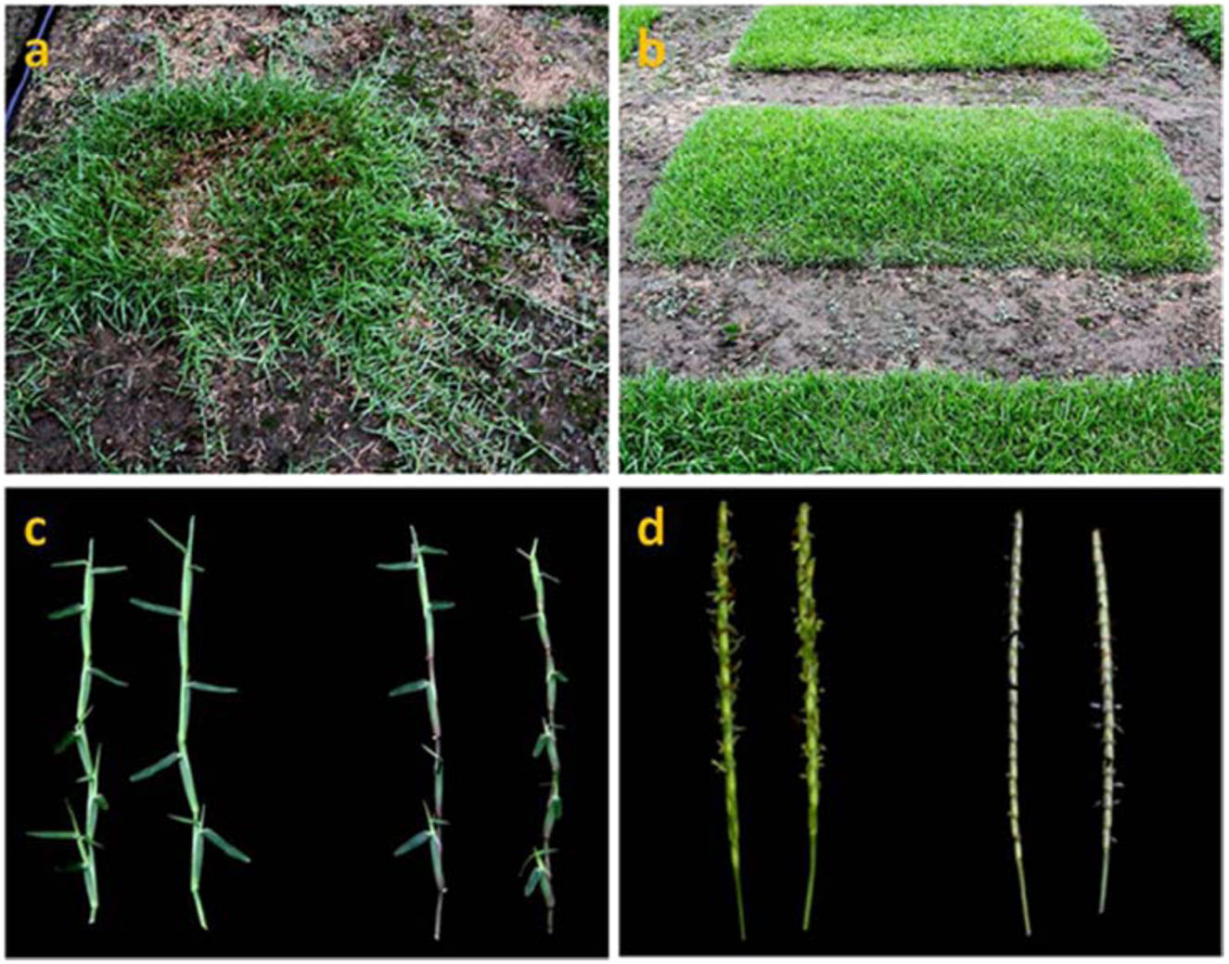
Photographs of centipedegrass showing a growing plug, a 2 by 1 meter (e.g., 2 × 1 m^2^) field plot, and two types of stolons and inflorescences. **a** Stolons growing from a plug in a field. **b** Overall appearance of the turf in the field. **c** Two types of stolons, namely, the green/yellow type and purple type. **d** Two different spikes, namely, a green/yellow spike and purple spike.

Centipedegrass [*Eremochloa ophiuroides* (Munro) Hack.] is a perennial warm-season (C_4_) grass that originated in China^1–3^. It is indigenous to Southeast Asia and is now widespread, with its distribution, including China (in the Yangtze River Basin and its southern area), Southeast Asia, the eastern and southern United States^4^, some parts of South America, the West Indies, and even parts of Africa^5^ and tropical northern and eastern Australia^6^. It has highly developed stolons during growth in the field and can form a dense canopy that provides continuous ground cover (Fig. 1). Centipedegrass is well known for its tolerance to aluminum and good adaptation to infertile soils and a wide range of climatic conditions^7,8^. It has great potential for commercial applications as a low-maintenance turfgrass, with advantages over other turfgrasses, such as lower management and fertilization requirements^7,9^. These properties of low maintenance, good adaptation to poor soil and a broad tolerance to biotic and abiotic stresses make centipedegrass a popular turfgrass across the southeastern United States and in the southern Yangtze River region of China^10,11^. The species is predominantly outcrossing and can be propagated vegetatively or by seed. Centipedegrass is widely used as turf for home lawns and recreational fields and for soil conservation in tropical and subtropical areas of the world;^9,12,13^ it can also be used as a feed supply for grazing livestock^7,14^. In addition, centipedegrass can potentially be used in environmental remediation due to its tolerance to and ability to absorb heavy metals^15,16^.

Genetic diversity is the key pillar of biodiversity within and between species and within ecosystems. Diversity in plant genetic resources (PGRs) provides an opportunity for plant breeders to develop new and improved cultivars with desirable characteristics^17^. Accordingly, the analysis of genetic diversity and variation in germplasm resources is an important part of turf breeding programs. Centipedegrass is one of the most important turfgrasses in tropical and subtropical regions, and the conservation, characterization, and utilization of its genetic diversity are becoming increasingly important in view of the high demand for high-quality turfgrass and global climatic changes. This demand is primarily because of the need to plant large areas of lawn in newly constructed modern residential areas and sports facilities in rapidly expanding cities in many developing countries.

Here, we present overviews of the results of major studies on the genetic variation in centipedegrass and of the recent progress made in describing centipedegrass diversity, mainly in the United States and China. The information summarized in the present article is of potential value not only for understanding the drivers of genetic variation in centipedegrass, but also for promoting the breeding and improvement of centipedegrass varieties using modern genetic and genomic tools.

## Taxonomy and cytogenesis

Centipedegrass belongs to the genus *Eremochloa* in the family Poaceae or Gramineae, subfamily Panicoideae, tribe Andropogoneae, and subtribe Rottboelliinae^18^. It is the only species in the *Eremochloa* genus that is used as turfgrass^12^. Centipedegrass is a C_4_ plant, similar to maize and sorghum^19,20^.

Centipedegrass is a diploid species with a somatic chromosome number of 2*n* = 2*x* = 18^2,21–24^ and a genome size of ~800 Mb^25^. Cytological observations of this species revealed that metaphase I of meiosis was mostly regular, with nine bivalents, but 1 to 15% of the microsporocytes had eight bivalents and two univalents due to a precociously dividing bivalent^2^. Centipedegrass is a sexually reproducing species with high-pollen stainability and normal sexual development involving megasporogenesis and an embryo sac, but it is self-incompatible^2,9^. An artificially induced tetraploid accession of centipedegrass can be generated by colchicine treatment, with an induction rate of 0.2%^26^. As polyploidization often results in the induction of chromosomal aberrations at the mitotic and/or meiotic level, such ploidy changes through artificial factors contribute to creating new centipedegrass germplasms, which are of high value in terms of sampling for cultivar improvement. However, cytogenetic information for natural accessions, as well as artificial lines of centipedegrass is currently limited. In addition, the above-mentioned findings were based on only a portion of centipedegrass samples, and such a small sample size might lead to an underestimation of the genetic diversity in this species. Therefore, it is necessary to use current cytogenetic approaches to carry out ploidy identification and karyotype analysis of more centipedegrass germplasms derived from different provenances or multiple breeding sources.

## Conservation of centipedegrass genetic resources

Practical breeding for high-quality and attractive turf stands relies mainly upon the germplasm resources available to breeders. The major centipedegrass collections are in China and the United States; most of the wild centipedegrass accessions in the United States were originally collected from China and are now maintained in the centipedegrass germplasm collection in Tifton, GA, and the USDA-ARS Plant Genetic Resources and Conservation Unit in Griffin, GA^1,7,13,24^. Currently, the Institute of Botany, Jiangsu Province and Chinese Academy of Sciences, which is one of the most important turf research institutes in China, has the only Engineering Research Center for Germplasm Innovation and Utilization of Warm-season Turfgrasses in the National Forestry and Grassland Administration (NFGA) and has the largest National Main Warm-season Turfgrass Gene Bank (NMWTGB) in China. The NMWTGB preserves centipedegrass genetic resources collected from different geographical regions in China and plant materials imported from the United States. To the best of our knowledge, the NMWTGB maintains the richest centipedegrass resources in the world, with ~192 accessions, including 11 cultivars initially registered and conserved ex situ (Table S1). Of these accessions, 148 are available for distribution as stolons or seeds. More than one-third of the registered accessions have been evaluated for plant morphological and economic characteristics, as well as phenological features and stress resistance characteristics. In addition, a core germplasm collection of 43 accessions was constructed in the NMWTGB based on heterogeneity and diversity analyses using sequence-related amplified polymorphism (SRAP) markers, which captured 98.74% of the allelic diversity in the entire collection of 148 accessions^27^. Considering all the deficiencies of a single marker type, the limited number of markers and the inadequate coverage of primitive germplasm resources, a more accurate and updated centipedegrass core collection urgently needs to be reconstructed by using the latest molecular marker techniques and additional collections from its distribution areas, especially those regions that were not visited during previous collection trips.

## Phenotypic diversity in centipedegrass

Centipedegrass is diverse in terms of its morphological and agronomic characteristics. Among all the phenotypic characters, stem node color (yellow and purple, as shown in Fig. 1) and stigma and anther colors show the most obvious variation among natural centipedegrass accessions^9,13^. In general, yellow stem type centipedegrass accessions show white stigmas and yellow anthers, while the purple stem type has stigmas and anthers that are uniformly purple or red^22^. Although limited morphological variation is present in natural centipedegrass accessions in the United States^9^, the presence of a wide range of genetic variation among the accessions in China has been found, expressed as variability in plant height^28,29^, stolon number and internode length^13,30^, leaf length^28,29^, number of inflorescences and diameter of internodes^28,30^, stem morphology (creeping vs. erect)^31^, seed yield^32^, and phenological phases, which mainly include the regreening stage, booting stage, blossom stage, maturity stage, wilting stage, and green period^33,34^. The narrower genetic base of centipedegrass collections in the United States than in China is likely because most of the centipedegrass accessions in the United States originated from a single collection introduced from China^9^. In addition, variations in some morphological characteristics related to lawn appearance, such as leaf length, plant height, and internode length and diameter, were also detected in centipedegrass hybrid generations^35,36^ (Table 1).

**Table 1:** Studies on phenotypic diversity in centipedegrass.

Germplasm or genotype		Sites^a^ (no)	Main variation in	References
Type	Number			
Natural accession	12	/	Stem and inflorescence color	Bouton et al.^22^
Natural accession	31	/	Limited morphological variation	Hanna^9^
Natural accession	15	7	Inflorescence density, plant height, leaf length	Bai et al.^28^
Natural accession	31	8	Stolon number and internode length	Liu et al.^13^
Natural accession	58	8	Fruit characters	Liu et al.^32^
Natural accession	59	8	Turf height, leaf length	Liu et al.^29^
Natural accession	36	5	Internode length and diameter	Liu and Liu^30^
Natural accession	40	5	Seed yield	Liu and Liu^32^
Natural accession	37	6	Phenological phases	Zong et al.^34^
Natural accession	60	13	Creeping and erect stem morphologies	Zhao^31^
Hybrids^b^	89	/	Leaf length, internode length, turf height and internode diameter	Zheng et al.^35,36^

^a^The sites represent different administrative and geographical divisions in China

^b^The hybrids were derived from a cross between a Chinese parent and a USA parent

Broadening the genetic base of centipedegrass is an important aspect of its breeding. To achieve this, one option is for many centipedegrass plants from different geographical sources to be randomly hybridized or for crosses between different genetic groups to be made to generate many populations with broad genetic variation. Alternatively, morphological variation in some leaf and stem features can be generated by gamma radiation, as was demonstrated by the development of a series of current cultivars, such as “AU Centennial”^37^ and “Tifblair”^38^.

## Molecular diversity in centipedegrass

The introduction of molecular biology techniques has contributed to the progress made in centipedegrass diversity research. In the past, centipedegrass diversity was mainly assessed using phenotypic data, as mentioned above. Recently, much effort has been made to analyze the genetic diversity in centipedegrass by using molecular approaches. The main work included revealing genetic variation in centipedegrass at the protein and DNA levels using protein markers (isozymes) and DNA markers (Table 2), respectively, as well as mining candidate genes responsible for desirable traits from RNA-seq data.

**Table 2:** Studies on molecular (genotypic) diversity in centipedegrass.

Germplasm or genotype			Technique	References
Type	Number	From		
Natural accession	12	USA	Protein markers	Bouton et al.^22^
Natural accession	5	USA	DAF	Weaver et al.^43^
Natural accession	5	China	Protein markers	Ren et al.^39^
Natural accession	15	China	Protein markers	Bai et al.^40^
Natural accession	9	China	AFLPs	Bai et al.^44^
Natural population	6	China	Protein markers	Xuan and Liu^41^
Natural accession	16	China	Protein markers	Liu and Liu^42^
Natural population	6	China	RAPD	Xuan et al.^45^
Natural accession	4	China	SRAPs	Zheng et al.^47^
Natural accession	60	China	ISSRs	Zhao et al.^46^
Natural accession	57	China, USA	SRAPs	Milla-Lewis et al.^10^
Natural accession	55	China, USA	SSRs	Harris-Shultz et al.^24^
F_1_ hybrids	89	China	SRAPs, EST-SSRs	Zheng et al.^50^
F_1_ hybrids	87	China	SRAPs, EST-SSRs	Wang et al.^51^
Natural accession	8	China	SRAPs	Guo et al.^48^
Natural accession	14	China, USA	EST-SSRs	Wang et al.^52^
Natural accession	43	China, USA	SSRs	Li et al.^49^

*DAF* DNA amplification fingerprinting, *AFLP* amplified fragment length polymorphism, *RAPD* random amplified polymorphic DNA, *SRAP* sequence-related amplified polymorphism, *ISSR* inter simple sequence repeat, *SSR* simple sequence repeat, *EST* expressed sequence tag

### Protein markers

Isozymes were among the most widely used markers for genetic variation analysis within and between populations before DNA markers were applied. Although they have now been largely superseded by more informative DNA-based markers, they were an excellent choice for projects that sought only to identify low levels of genetic variation. Early studies on isozymes showed different isoenzyme patterns of esterase and peroxidase among 12 U.S. centipedegrass accessions^22^. For centipedegrass collections or populations in China, several studies were also carried out on the variation in isozymes. Ren et al.^39^ tested peroxidase isozymes and esterase isozymes in five native centipedegrass samples from different areas of Sichuan and found that all five samples showed variations in the two enzymes. Bai et al.^40^ clustered 15 centipedegrass accessions collected from six geographical regions in China into two categories and four groups based on peroxidase isozyme analysis. By comparing peroxidase isozymes, esterase, and malate dehydrogenase isozymes among six populations of centipedegrass derived from geographical regions of mainland China, Xuan, and Liu^41^ evaluated the genetic diversity in centipedegrass populations and drew the conclusion that the genetic diversity of centipedegrass in China was more abundant within populations than among populations. Liu and Liu^42^ also revealed abundant genetic diversity in 16 centipedegrass accessions sampled from East China by electrophoretic analysis of peroxidase and esterase isozymes. However, the genetic diversity in centipedegrass revealed by these isozyme markers still needs to be further verified by more accurate DNA markers. In fact, portions of these research findings have already been validated or corrected in subsequent studies using DNA markers.

### Molecular markers

Molecular markers are short sections of DNA that differ among genotypes, and they are widely used for the identification of germplasm, assessment of genetic variation within and among populations, and determination of genetic relationships between various populations. The genetic relationships among five centipedegrass accessions (or cultivars) in the United States were assessed based on DNA amplification fingerprinting (DAF), and the relatively low levels of polymorphism among them suggested that these accessions are closely related and may share a common origin^43^. Similarly, genetic variations among accessions and populations of centipedegrass that were collected from different geographical regions in China were also analyzed in subsequent studies using AFLP^44^, RAPD^45^, ISSR^46^, SRAP^11,47,48^, and SSR markers^24,49^. The Nei’s genetic distances between nine centipedegrass collections derived from different geographic regions in China ranged from 0.135 to 0.994 for AFLP markers^44^, indicating broad genetic variation among these accessions. Significant variation was also detected in six centipedegrass populations consisting of 50 accessions by RAPD analysis^45^ and among 60 centipedegrass collections from 13 different geographical regions by ISSR analysis^46^, with a much higher level of genetic variation within populations than among populations. However, it is worth mentioning that a higher level of diversity in U.S. cultivars than in Chinese collections was revealed using SRAP and SSR markers; meanwhile, a lack of overlap in the ranges of diversity between the U.S. and most Chinese groups were found, and genetic diversity did not differ quantitatively between the U.S. and Chinese materials but did differ qualitatively^11^. Recently, Li et al.^49^ developed a large number of SSR markers based on the next-generation sequencing (NGS) approach and detected considerable genetic diversity among centipedegrass accessions by systematically analyzing the genetic variation among accessions in one core collection of 43 centipedegrass accessions. All the accessions were clustered into six groups according to genetic variation, while the genetic relationship among the 43 accessions did not correspond exactly to their geographical locations, which suggested that centipedegrass experienced outward expansion from its origin site, leading to a complex genetic background and evolutionary history. These analyses depicted a high level of polymorphism within cultivars and a dramatic difference in diversity between/among centipedegrass populations.

Owing to the high level of heterozygosity of centipedegrass plants, research progress on genetic map construction and quantitative trait locus (QTL) mapping of major economic traits in centipedegrass has been slow. The first genetic linkage maps of centipedegrass were constructed using SRAP and SSR markers and were based on 89 F_1_ progenies of a cross between two centipedegrass ecotypes (E102 and E092-1). The maps consisted of a female linkage map with 89 loci (85 SRAPs and 4 EST-SSRs) and a male linkage map with 71 assigned SRAP loci^50^. The same research group subsequently produced another set of genetic maps using the same molecular markers from a population of 87 F_1_ plants derived from crossing two different ecotypes (E142 and E022) and detected a total of 14 QTLs on the nine linkage groups of the maps; the QTLs were associated with seed yield, vegetative traits and cold tolerance, and could explain 8.71–23.61% of the phenotypic variation^51^. With the rapid development of high-throughput sequencing technology, additional specific molecular markers for centipedegrass can be rapidly developed at low cost. Therefore, constructing high-density genetic maps in centipedegrass will be an important goal for future research and has the potential to identify the genes responsible for traits of interest.

### Transcriptomics and genomics

To date, the transcriptomes from one cultivar and two accessions of centipedegrass have been sequenced^49,52^. For the cold-tolerant variety “TifBlair”, a normalized transcriptome library was prepared and sequenced from leaves of seedlings, resulting in a transcriptome with 45,575 unigenes and 84,781 contigs^52^. A second normalized library was constructed from various centipedegrass tissues, including leaf, stolon, and spike tissues, resulting in more gene clusters^49^. Furthermore, a comparative RNA-seq study revealed 329 putative gene products differentially expressed between red-purple and green stolons and 829 differentially expressed genes between red-purple and yellow-green spikes^49^. Owing to various environmental adversities during lawn growth, future research endeavors should be devoted to the transcriptomic analysis of centipedegrass in response to cold and drought stresses and of its tolerance to barren and acidic soils, among other environmental stressors.

With the rapid development and wide applications of NGS technologies, genomic research on several grass species has recently moved from small-scale analysis of genetic polymorphisms toward whole-genome sequencing and genome-wide polymorphism surveys. The availability of genomic sequences for a turfgrass species will greatly increase the number of genome-scale investigations of fundamental mechanisms of resistance to biotic and abiotic stresses. However, to date, there have been no reports on centipedegrass whole-genome sequencing or related genome-sequencing projects, while a few such studies have been performed in zoysia^53^ and perennial ryegrass^54^ via NGS techniques.

## Centipedegrass diversity in certain stress-related traits

### Drought tolerance

Drought stress often occurs during the process of lawn growth and management, and it results in a decline in the quality and productivity of turfgrasses. Studies investigating the effect of moisture deficits on the performance of centipedegrass plants ranged from studies of variability in key morphological characters and responses^55–60^ to studies of physiological traits related to turf quality^61–66^. However, systematic studies on genetic variability in drought tolerance among centipedegrass genotypes have rarely been performed, which severely restricts the breeding of drought-tolerant centipedegrass varieties. Nevertheless, there have been a few successful attempts to produce drought-resistant mutants by radiation-induced mutation in centipedegrass seeds^67–69^ and by culture-induced variation in somatic embryogenesis^70^. These mutant plants showed greater tolerance to drought stress and thus could be potential germplasm resources for breeding drought-tolerant centipedegrass. Hence, it will be important to understand genetic variability in drought tolerance within the centipedegrass gene pool in the future.

### Salinity tolerance

Salinity is a detrimental abiotic stressor for turfgrass plant growth in salt-affected soils. Some artificially bred cultivars of centipedegrass show intraspecific variations in morphological characteristics and turf quality related to salinity tolerance; for example, there is a difference in salt tolerance between “TifBlair” (a salt-tolerant cultivar) and “Common” (a salt-sensitive cultivar). Studies on physiological responses to salt stress have revealed significant differences between salt-tolerant and salt-sensitive cultivars/accessions^71,72^. Generally, however, centipedegrass is a salinity-sensitive species, in contrast to other warm-season turfgrasses^19,73–77^.

### Aluminum and acidity tolerances

Aluminum (Al) toxicity is a major limiting factor for plants growing in acidic soils. Wild centipedegrass is mainly distributed in acidic soils in tropical and subtropical regions, so investigating and documenting the effect of such soil conditions on centipedegrass production and productivity is worthwhile. It has been widely assumed that centipedegrass is well adapted to acidic soils and has excellent Al resistance. A high degree of genetic variation in Al tolerance in centipedegrass collections in China, as well as in the cultivars “TifBlair” and “Common”, was found based on plant growth and physiological responses to Al^78–80^. A preliminary genetic analysis of aluminum tolerance revealed that aluminum tolerance in centipedegrass is not controlled by a single major gene^81^. Root exudation of citric acid is one of the key mechanisms for Al tolerance in centipedegrass^82^.

### Cold tolerance

Centipedegrass is sensitive to low temperatures, is vulnerable to freezing injury, and has a poor overwintering ability. Thus, its adaptation is limited to areas with mild winter temperatures. An early study revealed no measurable differences in cold resistance among centipedegrass plants from different sources in the United States^83^. In contrast, several centipedegrass accessions in China showed great differences in cold tolerance^84,85^. The freezing tolerance of centipedegrass was positively correlated with the levels of polyamines (Pas) and antioxidants, the levels of proline and soluble carbohydrates, and the ratio of soluble carbohydrates to starch^86–89^. The inheritance of cold tolerance in centipedegrass is additive and dominant with epistatic effects and is controlled by two major genes^90^. To date, one QTL for cold tolerance has been identified by constructing a group of genetic linkage maps of centipedegrass^91^. Recently, several centipedegrass mutants have been produced by radiation^69,86^ and by somaclonal variation^91,92^, and the resultant mutants show good cold resistance. These mutants are valuable materials for both centipedegrass research and centipedegrass breeding purposes.

## Conclusions and future perspectives

The value of germplasm resources is determined by their genetic diversity, availability, and utility. The wide spectrum of genetic diversity in growth phenotypes and stress-related traits in centipedegrass implies great opportunities for genetic improvement through either direct selection or hybridization between parental lines with desirable traits. Cytological and molecular methods have provided powerful tools for a deeper analysis of genetic variation in centipedegrass. Some mutagenized materials have been developed to supplement the natural diversity present in centipedegrass. As in the many studies that were reviewed here, many efforts have been made over the past few decades to estimate and utilize the genetic variation in centipedegrass populations grown in the United States. Nevertheless, due to the limited populations and genotypes of U.S. accessions, the actual diversity is not reflected in the available centipedegrass germplasm. Although wild resources of centipedegrass are abundant in China and have many unique and useful characteristics, such as a diversity of turf performance and levels of stress tolerance, the exploitation and utilization of resources based on their genetic diversity are still limited. Therefore, it is necessary and valuable to further explore the diversity of centipedegrass germplasms that are currently growing in China and other places in South Asia. Such further germplasm exploration and evaluation may lead to the identification of additional improved centipedegrass materials, which could be conducive to the expanded production of high-value turf. Molecular cytogenetic and new sequence-based approaches will provide opportunities for in-depth studies of the genetic variation and phylogenetic relationships among global collections of this species. In view of the above facts, future research should emphasize four aspects: (1) further collection of centipedegrass germplasm from all of its distribution areas, and especially from regions not sampled during previous collection trips, in order to construct a richer and more complete germplasm bank and an updated core collection; (2) cytogenetic studies on the chromosome structure and function of centipedegrass both for breeders and for other scientists interested in the analysis and manipulation of breeding material at the chromosomal level; (3) studies of genomics, transcriptomics, proteomics, and metabolomics in centipedegrass using high-throughput sequencing techniques; and (4) mining and functional research on the genes responsible for desirable traits such as strong aluminum tolerance and good tolerance to infertile soils, which will ultimately aid in cultivar improvement. With this article, the authors hope that the concerted efforts of all stakeholders in research, development, and funding can be united globally to promote the healthy development of the turf industry.

## Supplementary information

Table S1
